# Cardiomyocytes and Macrophages Discourse on the Method to Govern Cardiac Repair

**DOI:** 10.3389/fcvm.2018.00134

**Published:** 2018-10-02

**Authors:** Ingrid Gomez, Vincent Duval, Jean-Sébastien Silvestre

**Affiliations:** Institut National de la Santé et de la Recherche Médicale (INSERM), UMRS-970, Paris Centre de Recherche Cardiovasculaire, Université Paris Descartes, Sorbonne Paris Cité, Paris, France

**Keywords:** heart, inflammation, macrophages, heart failure, myocardial infarction

## Abstract

In response to pathophysiological stress, the cardiac tissue undergoes profound remodeling process that incorporates the elimination of dying resident cells, compensatory hypertrophy of functional cardiomyocytes, growth and remodeling of the vascular compartment and formation of a fibrotic scar. Accumulating evidences indicate that cardiac remodeling is, at least in part, controlled by a complex crosstalk between cardiomyocytes and macrophages. The strategic location of abundant macrophages to the proximity of cardiomyocytes suggest that they could regulate the fate of cardiomyocytes in the injured heart. As such, macrophages appear as critical support cells for cardiomyocytes and play central roles in cardiac hypertrophy, fibrosis and remodeling. Notably, the cardiac tissue expands heterogeneous population of cardiac macrophages through local proliferation of resident macrophage as well as recruitment and differentiation of blood-derived monocytes. It has also been suggested that cardiac-resident macrophages display distinct functional properties from that of monocyte-derived macrophages in cardiac tissue. Furthermore, macrophages are an overflowing source of biological entities with non-canonical roles on cardiac conduction or cardiomyocyte proliferation by regulating action potential diffusion or cardiac cell cycle reentry. Alternatively, stressed cardiomyocytes can trigger the release of a broad repertoire of instructive signals that can regulate macrophage number, skew their phenotype and therefore direct their beneficial or deleterious actions. In this review, we highlight recent discoveries describing how the intricate dialogue between cardiomyocytes and macrophages can shape the deleterious or healing signaling mechanisms in the injured cardiac tissue.

Pathological conditions (hypertension, heart valve disease, myocardial infarction, cardiomyopathy) result in cardiomyocyte hypertrophy and cardiac remodeling progression associated with systolic and diastolic dysfunction. This adverse ventricular remodeling precipitates the occurrence of heart failure, arrhythmia, or sudden death. Hypoxia, neuro-humoral activation, and mechanical stress initiate multiple signaling pathways leading to cardiomyocyte death and hypertrophic growth of surviving cardiomyocytes. Nevertheless, the heart also entails different types of non-myocyte cells, including vascular cells, fibroblasts and inflammatory cells. Notably, emerging evidences indicate that cardiac remodeling is regulated by direct and indirect communications between cardiomyocytes and inflammatory cells. In response to pathophysiological stress, the abundance of inflammatory cells and particularly, that of macrophages, and their proximity to cardiomyocytes, position them to critically regulate cardiomyocyte homeostasis in the injured heart. As such, macrophages appear as critical support cells for cardiomyocytes and play central roles in cardiac hypertrophy, fibrosis and remodeling. In this review, we highlight recent advances, mainly related to experimental observations, in our understanding of the decisive interaction between macrophages and cardiomyocytes.

## Origin of cardiac macrophages

During early gestation, around embryonic day 7, primitive hematopoiesis occurs and macrophages expand in the extra-embryonic yolk sac. Subsequently, erythromyeloid precursors emerge from the yolk sac, and generate fetal macrophages. The onset of vascularization is associated with erythromyeloid precursors migration to the fetal liver. Concomitantly, hematopoietic stem cells (HSCs) arise from the aorta-gonad-mesonephros and migrate to the fetal liver. In the perinatal period, bone marrow serves as a major reservoir of HSCs and synthesizes the complete repertoire of immune cells (1).

The development of genetic fate-mapping techniques allows to classify and track distinct macrophage subsets, and when associated with parabiotic and adoptive-transplant studies, permits to discern the origin of tissue macrophages. Using this combination of methods, it has become obvious that most of cardiac macrophages derives from precursor of embryonic origin, but not from circulating monocytes after birth (2). Murine cardiac macrophages (CD45^+^CD11b^+^F4/80^+^CD64^+^ or CD68^+^) are further categorized by expression of CCR2 (C-C chemokine receptor type 2), MHC-II (major histocompatibility complex II), and the lymphocyte antigen 6 complex (Ly6C). High and low expression of Ly6C can be used to discriminate inflammatory and reparative macrophages but the use of both CCR2 and MHC-II is useful to distinguish three separate and discrete cardiac macrophages pools from different origins. In steady state, the adult heart contained two resident macrophages subsets, MHC-II^low^CCR2^−^ and MHC–II^high^CCR2^−^ cells. These macrophages are separate from the blood monocyte pool and originate mainly from yolk sac- and fetal liver- embryonic progenitors (2, 3). The third macrophage pool expresses CCR2 (MHC-II^high^CCR2^+^) but is smaller numerically and is derived entirely from blood monocytes (2, 3). In the steady state, tissue-resident macrophages constitute the main subset of cardiac macrophages and are preserved through tissue proliferation (2). Nevertheless, it has been suggested that self-renewal of cardiac-resident macrophages declines with aging and that a substantial pool of cardiac resident macrophages is replenished by monocyte-derived macrophages, even in the lack of pathological triggers and inflammation (4). Indeed, using alternative gating strategy based on MHC-II and CX3CR1 expression, it has been shown that the proliferation capacity of embryo-derived cardiac macrophages is progressively reduced from the peri-natal period (4).

Of note, comprehensive transcriptional analysis of resident macrophages suggests that resident macrophages isolated from various organs are transcriptionally different from each other (5, 6). It is therefore likely that the tissue niche provides instructive signals coordinating macrophage phenotype and that the cardiac environment shapes the gene repertoire and controls macrophage-related functions. Furthermore, alteration of this cardiac niche is expected to affect the transcriptional network of cardiac macrophages resulting in a continuum of polarization states in the pathological heart.

**Figure 1:**
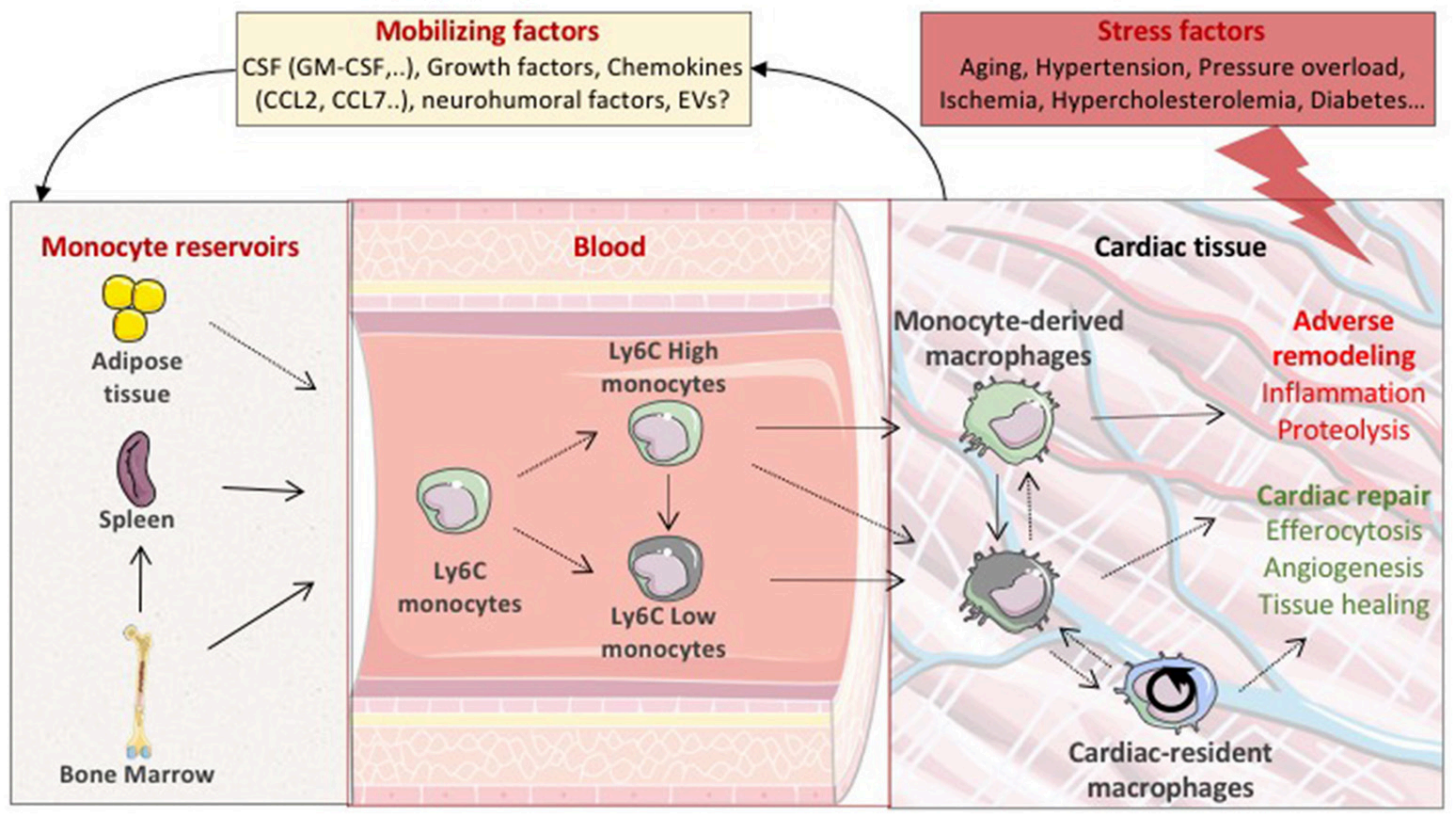
Life cycle of macrophages during cardiac stress. In pathological settings, the stressed cardiac tissue sends mobilizing factors to monocyte reservoirs including bone marrow and spleen, leading to the emergence of two principle monocyte subsets depicted by the expression of Ly6C (Ly6C High or Low). Circulating Ly6C positive monocytes are then recruited into the injured cardiac tissue and differentiate to macrophages. Local proliferation of cardiac resident macrophages also participates to the expansion of cardiac macrophage population. Cardiac-resident macrophages and monocyte-derived macrophages are expected to display distinct functional properties precipitating or preventing adverse ventricular remodeling. CSF, colony-stimulating factors; GM-CSF, granulocyte macrophage colony stimulating factor; EVs, extracellular vesicles.

Physiological and pathological settings adjust the number of each cardiac macrophage pools. Macrophage number could depend on local expansion of resident macrophage or recruitment and differentiation of blood-derived monocytes. In the absence of circulating monocytes, such as in CCR2 deficient mice, resident cells can repopulate the pool of cardiac macrophages. After transient depletion of macrophages with clodronate liposomes, circulating monocytes infiltrate the myocardium and are able to differentiate into long lasting populations of cardiac macrophages. Furthermore, CCR2– macrophages disappeared after a cardiac insult and blood monocytes fully replenish heart macrophages (2, 7). Hence, after a cardiac insult, it is likely that bone marrow-derived monocyte populations represent the major cardiac macrophage substitute. Notably, in the bone marrow, a CCR2^+^CD150^+^CD48^−^ Lineage^−^Sca-1^+^c-Kit^+^ hematopoietic subset has been identified as the main precursor of myeloid cells after ischemic insult (8). The cardiac tissue produces mobilizing factors such as granulocyte/macrophage colony-stimulating factor or CCL (CC motif chemokine ligand)2 and CCL7 that act distally to foster monocyte mobilization from the bone marrow to the blood (9–11). Bone marrow-derived HSCs lead to the emergence of two principle monocyte subsets depicted by the expression Ly6C. Classical Ly6C^high^ monocytes appear to originate from Ly6C^+^ monocyte progenitors, and non-classical Ly6c^low^ monocytes, which likely differentiate from Ly6C^high^ monocytes, through a Nr4a1-dependent transcriptional program (12–14). Bone marrow derived Ly6C monocytes are recruited into the injured cardiac tissue and differentiate to macrophages. In addition to the bone marrow, the spleen also acts as a reservoir for Ly6C^high^ monocytes and participates to cardiac macrophage repopulation [(15); Figure 1]. One can also speculate that additional compartment could participate to the inflammatory cell influx within the cardiac tissue. In this line of reasoning, the pericardial adipose tissue has been shown to contain a high density of lymphoid clusters (16). The white adipose tissue has also been identified as a reservoir of mast cell progenitors, that do not originate from the bone marrow, and are able to home to cardiac tissue through a stem cell factor dependent signaling (17). The putative contribution of adipose tissue to the monocyte and/or macrophage pool remains to be defined. Altogether, these results indicate that, following the disruption of homeostasis; multiple complementary pathways could participate to the dynamic regulation of the heterogeneous population of cardiac resident macrophages.

In macrophage-depleted mice, infarct healing is impaired, and myocardial rupture occurs suggesting a net beneficial role of the heterogeneous population of cardiac macrophages in cardiac repair (18–20). Resident macrophages are expected to display functional properties distinct from that of monocyte-derived macrophages. Transcriptional analysis revealed that monocyte-derived macrophages synchronize cardiac inflammation, but are less efficient in antigen processing and clearance of dying cells (2). In particular, CCR2^+^ macrophages contain distinct components of the NLRP3 inflammasome and are instrumental in IL-1β release (2). On the same note, chemoattractants CXCL (C-X-C motif chemokine ligand)2 and CXCL5 produced by CCR2^+^ resident macrophages have been shown to specifically contribute to the initial neutrophil extravasation into the ischemic area (21). Inhibition of monocyte and monocyte-derived macrophage recruitment improves outcome after acute MI and myocardial infarction-induced heart failure. Conversely, cardiac-resident macrophages (MHC-II^low^CCR2^−^ and MHC-II^high^CCR2^−^) seem to display robust proangiogenic and mitogenic properties, suggesting a reparative potential for these macrophage subsets [(22); Figure 1].

Nevertheless, it must be acknowledged that there is no consensus to harmonize the phenotypic classification of macrophages, which induces uncertainty in the characterization as well as function of the distinct sub-populations of cardiac macrophages. An ambiguity amplified by the use of enzymatic digestion methods designed to isolate cardiac inflammatory cells that alter the levels of cell-surface markers and by the shared expression of some markers such as CD11b, Ly6C or F4/80 on dendritic cells or myeloid derived suppressor cells (23).

Although macrophages are clearly present in human heart (24), information regarding their function, origin and heterogeneity in steady state and pathological conditions are lacking. Nevertheless, two distinct populations of macrophages have been identified in left ventricular myocardial specimens from patients with dilated and ischemic cardiomyopathies (25). CCR2+ and HLA-DR High (the human homolog of MHC II) macrophages are monocyte-derived cells with specific pro-inflammatory potential whereas CCR2– and HLA-DR High macrophages are resident cells with reparative properties [(25); Table 1].

**Table 1:** Immunophenotypic properties of different subsets of embryo- and monocyte-derived cardiac macrophages.

		**Origin of cardiac Mφ**	**Inflammatory cell signature markers**	**Mφ signature markers**	**Specific signature markers**
Mice	Gating strategy 1	Embryo-derived Mφ	CD45+, CD11b+, Auto+, Ly6C–	CD64+, Mertk+, F4/80+	MHCII High/Low and CCR2–
		Monocyte-derived Mφ	CD45+, CD11b+, Auto+, Ly6C–	CD64+, Mertk+, F4/80+	MHCII High and CCR2+
	Gating strategy 2	Embryo-derived Mφ	CD11b+, Ly6C Low, CD11c Low-Int	CD14+, CD64+, F4/80+, Mertk+	CX3CR1+ and MHCII–
		Monocyte-derived Mφ	CD11b+, Ly6C Low, CD11c Low-Int	CD14+, CD64+, F4/80+, Mertk+	CX3CR1± and MHCII+
Human	Gating strategy 3	Resident Mφ	CD45+	CD14+, CD64+, Mertk+, CD68+	CCR2– and HLA-DR High
		Monocyte-derived Mφ	CD45+	CD14+, CD64+, Mertk+, CD68+	CCR2+ and HLA-DR High

*Gating strategy 1, 2, and 3 from Epelman et al. (2), Molawi et al. (4), and Bajpai et al. (25), respectively. Mφ indicates macrophages; Auto, auto-fluorescence; int, intermediate*.

## Macrophages and outside-in signaling within cardiomyocytes

### Canonical role of macrophages

**Figure 2:**
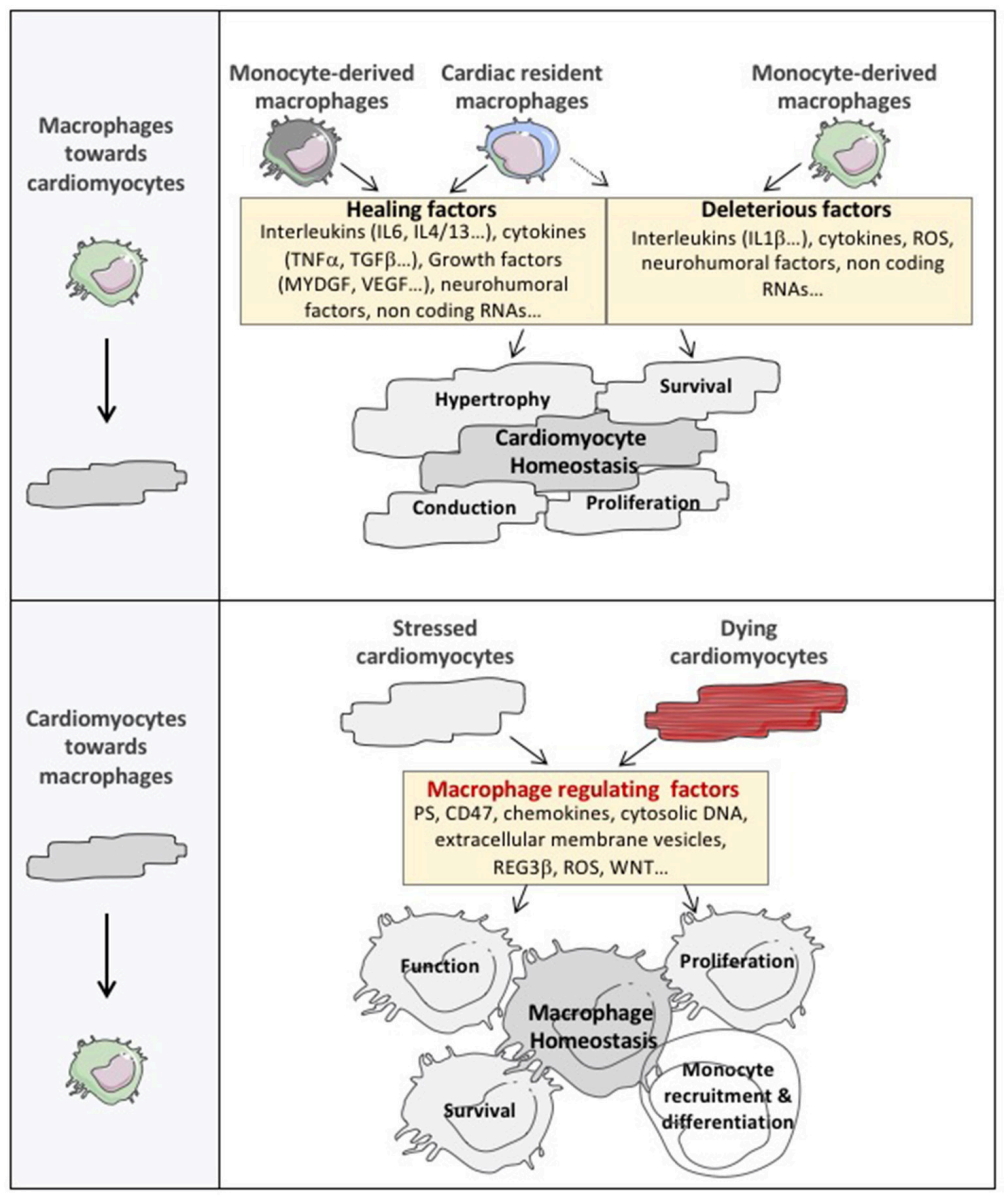
The cardiomyocyte and macrophage cross-talk in cardiac tissue. The expanded cardiac macrophage population plays an important role in the regulation of cardiac dysfunction by secreting factors that directly or indirectly alter cardiomyocyte homeostasis. Notably, considering their abundance, diversity, and phenotypic plasticity, macrophages represent a major reservoir of factors controlling cardiomyocyte hypertrophy, survival and contractility. Furthermore, cardiac macrophages exert non-canonical roles on cardiac conduction or cardiomyocyte proliferation by regulating action potential diffusion or cardiac cell cycle reentry. Nevertheless, the communication between cardiomyocytes and macrophages operates in both directions and cardiomyocytes dialogue toward cardiac macrophages to drive their number, function and phenotype through the release of a broad catalog of macrophage regulating factors. MYDG, myeloid derived growth factor; PS, phosphatidyl serine; ROS, reactive oxygen species; DNA, deoxyribonucleic acid; REG3β, regenerating islet-derived protein 3-beta.

The interaction between macrophages and cardiomyocytes hypertrophy and survival has been studied in experimental model of acute injury (mainly acute myocardial infarction) or heart failure following pressure overload, hypertensive treatment, or myocardial infarction. Whatever the incentive stimulus, the failing heart contains increased number of macrophages that expand by both local proliferation of resident macrophage and further recruitment and differentiation of circulating monocyte (2, 7, 26, 27). Interactions of cardiac macrophages with other non-myocyte cells including inflammatory cells, vascular cells and fibroblasts shape the dysfunctional heart. These cardiac macrophages also secrete factors that directly or indirectly alter the cardiac extracellular matrix network. Hence, the net impact of cardiac macrophages on cardiomyocyte likely relies on pleiotropic effects involving regulation of distinct and complementary processes. Furthermore, the molecular cascades involved in macrophage specific effects on cardiomyocytes are poorly understood. Nevertheless, the cardiac influx of macrophages on pressure overload, for example, precedes signs of hypertrophy and heart failure, suggesting a direct contribution to cardiomyocyte hypertrophy and survival [(28); Figure 2].

Considering their abundance, diversity, and phenotypic plasticity, macrophages represent a major reservoir of cytokines. Inflammatory signals induce interleukin(IL)6 release by macrophages and neutralizing IL-6 reduces cardiomyocyte hypertrophy (29, 30). Acutely, IL-6 also prevents cardiomyocytes against oxidative stress-induced cellular damages. IL-6 receptor cooperates with at least one subunit of the signal-transducing protein gp130. Of note, complete loss of gp130 results in massive induction of myocyte apoptosis and dilated cardiomyopathy in response to pressure-overload (31). Alternatively, in chronically exposed myocytes, IL-6 family signaling reduces the basal contractility and the beta-adrenergic responsiveness of the myocytes (32).

Specific subtype of pro-inflammatory CCR2 positive macrophages is a major purveyor of IL-1β (2). Interestingly, in a phase 3 trial, the use of canakinumab, an IL-1 β neutralizing antibody, has been shown to decrease the incidence of repetitive atherothrombotic event in patients with myocardial infarction already on state of the art treatment (33). Inflammatory macrophages also release IL-18, another member of the IL-1 family (34, 35). IL-18 enhances cardiomyocyte hypertrophy and exacerbates cardiac dysfunction as well as fibrosis in infarcted and non-infarcted heart (36, 37). IL-18 could also precipitate adverse ventricular remodeling by reducing capillary density in the ischemic tissue (38). Toll-like receptor activation induces the production of tumor necrosis factor (TNFα) from macrophages. In a genetic heart failure model, TNF-α has been shown to confer cardioprotection by maintaining normal intercalated disc structure and mitochondrial integrity as well as cardiomyocyte function (39). Transforming growth factor (TGF)β produced by cardiac macrophages triggers extracellular matrix deposition (40) but is also a direct mediator of the hypertrophic growth response of the heart (41). Myeloid-derived growth factor is secreted by macrophages and stimulates cardiomyocyte survival (42). In addition, macrophages synthesize IL-10 and leukemia inhibitory factor, which subsequently abrogate apoptosis of hypoxic cardiomyocytes (43).

Beyond cytokine production, macrophages are an overflowing source of biological entities with pro-hypertrophic and survival potential. For example, Galectin-3, a macrophage-derived mediator, contributes to cardiac hypertrophy (44). Removal of micro(mi)RNA-155 in macrophages reduces cardiac hypertrophy and cardiac dysfunction following pressure overload (28). Mineralocorticoid receptor deletion in macrophages abrogates cardiac hypertrophy after aortic constriction (45) or in the setting of hypertension (46, 47). Mice with macrophage harboring prolyl hydroxylase domain protein 2 deficiency display attenuated cardiac hypertrophy and contractile dysfunction after hypertensive treatment (48).

### Non-canonical role of macrophages

#### Cardiac conduction

Recent work also reveals that resident cardiac macrophages establish direct connections to cardiomyocytes via connexin 43 gap junctions in the atrioventricular node. *In vitro*, neonatal cardiomyocytes cause rhythmic depolarizations in macrophages, which in turn, alters the electrophysiological properties of cardiomyocytes. Since macrophages are poorly polarized cells, their coupling with cardiomyocytes depolarizes them. While in the “working” cardiomyocytes an increase of the resting potential inactivates the sodium channels and slows the conduction, the depolarization of the atrioventricular node cardiomyocyte depends on the calcium channels, whose voltage-dependent inactivation is shifted toward higher positive voltages. Activation of a rhodopsin 2 channel specifically expressed in macrophages causes sodium entry and depolarization, improving nodal conduction. Conversely, inactivation of connexin 43 specifically in macrophages, or the congenital deficiency of macrophages, affect the propagation of cardiac electrical impulses, attesting to the existence of a functional connection between resident macrophages and specialized cardiomyocytes within the nodal tissue (24). Macrophages could also regulate conduction abnormalities beyond the atrioventricular node, including the course of atrial fibrillation or ventricular arrhythmias induced by ischemia. Clinically, atrioventricular block is a common indication for implantation of cardiac pacemakers, but many cases of atrioventricular block occur for unknown reasons. Macrophage phenotype and number are modified after acute myocardial infarction and heart failure, conditions associated with sudden cardiac death and ventricular arrhythmias. In this regard, recent work has shown that diabetic inflammation causes secretion by resident macrophages of IL-1β, which destabilizes the electrical activity of cardiomyocytes potentially promoting the occurrence of ventricular arrhythmias (49). Other cardiac inflammatory diseases, including Chagas disease, Lyme disease, and myocarditis, initiate conduction defects. In these pathological conditions, cardiac macrophages could also participate to the development of atrioventricular block in addition to cardiomyocytes and specialized conductive tissue deregulations. Conduction system development or genetic abnormalities could also be dependent of cardiac macrophage-related actions.

#### Cardiomyocyte regeneration

Change in monocyte/macrophage gene expression is associated with decrease in cardiomyocyte proliferation with aging (50). In the neonatal heart, clodronate liposome-induced phagocytic cell depletion abrogates heart regeneration. Human fetal and adult mononuclear phagocytes show distinct pro-inflammatory features after LPS stimulation (2). Furthermore, it has been shown that in response to injury, resident CCR2– reparative cardiac macrophages dominate in the neonatal cardiac tissue and directly foster cardiomyocyte proliferation (22). Comparative analyses have been performed in many species revealing that the regenerative capacity of the cardiac tissue sparsely exists across the animal kingdom, and seems to correlate with immature immune system. As an example, zebrafish exhibits unchallenged ability to regenerate cardiac tissue. Interestingly, another teleost, the medaka, does not share this capacity. Comparative transcriptomic analyses following cardiac cryoinjury in Zebrafish and medaka point to major differences in immune cell dynamics between these two models. Delayed macrophage recruitment in zebrafish hampers cardiomyocyte proliferation and scar resolution. Delayed and reduced macrophage recruitment is observed in medaka, along with inefficient cardiac regeneration. Stimulating Toll-like receptor signaling in medaka activates immune cell dynamics and galvanizes cardiomyocyte proliferation and scar resolution (51). During the regenerative process, cardiomyocytes undergo a transcriptional reversal of cell differentiation program characterized, at least in part, by recrudescence of embryonic gene expression. Interestingly, IL-13 has been identified as a potential upstream regulator of the core network, which induced cardiomyocyte cell cycle entry, in part by the STAT3 pathway (52). Altogether, these studies provide further insight into the complex role of the immune response during cardiac repair, and suggest that macrophage could serve as a platform to release cytokines and growth factors, promoting cardiomyocyte proliferation, at least in neonatal mammalian heart.

## Cardiomyocytes and outside-in signaling within cardiac macrophages

The communication between cardiomyocytes and macrophages operates in both directions and cardiomyocytes dialogue toward cardiac macrophages using different languages (Figure 2).

First, dying cardiomyocytes send some “eat me” signals to stimulate clearance of dying cells and prevent collateral myocyte loss and infarct expansion. Efficient phagocytic clearance of cardiac apoptotic cells by macrophages involved the coordinated role of 2 major mediators of efferocytosis, the myeloid-epithelial-reproductive protein tyrosine kinase (MerTK) and the milk fat globule epidermal growth factor 8 (MFG-E8). Macrophage defective for efferocytosis leads to cardiac dysfunction (53, 54). Interestingly in ischemic heart, dying cardiomyocytes send unappropriated signals, such as upregulation of integrin-associated protein CD47 or shedding of macrophage MerTK, that impair phagocytic removal by cardiac macrophages (55, 56). Notably, resident MHCII^low^CCR2^−^ cells are characterized by elevated expression of MerTK and, release pro-reparative cytokines, such as TGF-β, in response to apoptotic cell engulfment (57). Of interest, non-professional phagocytes including cardiac myofibroblasts, have also been shown to efficiently engulf dead cells in the infarcted heart. These cardiac myofibroblasts secrete MFG-E8 promoting efferocytosis and acquisition of anti-inflammatory properties (58). Communication between professional and non-professional phagocytes likely coordinates phagocytosis of dying cells in the vicinity of the damage (59). As a prototypic example, in epithelial tissue, following clearance of apoptotic cells, macrophages release soluble growth factors, such as insulin-like growth factor 1, which redirect the phagocytosis of neighbor epithelial cells toward uptake of smaller vesicular components (60). Nevertheless, the existence of this crosstalk between macrophages and non-professional phagocytes remains to be detailed in the pathological cardiac tissue.

Second, in pathological conditions, activated cardiomyocytes can release a broad panel of instructive signals that could control macrophage number and skew their phenotype. For example, neutrophils and macrophages produce the cytokine oncostatin M, which galvanizes the cardiomyocytes to produce the regenerating islet-derived protein 3-β. Subsequently, regenerating islet-derived protein 3-β improves monocyte-derived macrophage number in the cardiac tissue (61). Other molecular entities delivered by hypoxic cardiomyocytes such as inhibitors of the WNT pathway, radical oxygen species or the release of cytosolic nucleic acid also modulate the number and phenotype of cardiac macrophages (62–64). In hypertensive heart failure model, microarray and immunohistochemistry analysis revealed a cardiomyocyte specific upregulation of 12/15-lipoxygenase, a major enzyme metabolizing arachidonic acid into hydroxy-eicosatetraenoic acids. Cardiomyocyte overexpression of 12/15-lipoxygenase increases cardiac CCL2 contents leading to accumulation of inflammatory macrophages and systolic dysfunction (65). Extensive amount of evidences highlights that cell-to-cell communication involves extracellular vesicles (EVs). EVs regulate major biological functions, including inflammation. Presence of EVs in cardiomyocyte cytoplasm has been revealed in human hearts. Culture cardiomyocytes are also able to release EVs *in vitro* (66). Acute myocardial infarction in mice transiently enhances EV levels in the left ventricle. Both large (252 ± 18 nm) and small (118 ± 4 nm) EVs are produced and mainly emerge from cardiomyocytes. Of note, large EVs, but not small EVs, are able to control IL-6, CCL2, and CCL7 release from cardiac monocytes (67). Hypoxic cardiomyocytes have also been shown to release high rate of small EVs (mainly exosomes) containing excessive expression of TNF-α (68). Mixt EV population or small EVs (mainly exosomes) have emerged as critical agents of cardiac repair triggered by different type of cell therapy (69–71). EV-related effects could be mediated by cell-surface receptor activation or delivery of multiple components within the targeted cells. Notably, small EV isolated from cardiosphere-derived cells are enriched in several miRNAs, such as miR-181b, and have been shown to specifically target CD68-positive cardiac macrophages switching their phenotype toward a reparative state (72). Cardiac EVs have also been identified in fragments of the interventricular septum obtained from patients undergoing extracorporeal circulation for aortic valve replacement suggesting that cardiomyocyte-derived EVs could constitute a major component of the cardiomyocyte/macrophage crosstalk (67). One can also speculate that these cardiac EVs could reach the bone marrow and/or the spleen, where they could stimulate a distinct myeloid-biased progenitor subset or monocyte mobilization. Nevertheless, the specific role of large or small cardiac EVs on different subset of inflammatory compounds as well as characterization of their core signaling mediator need to be further explored. Finally, the mechanical deformation of cardiomyocytes by pathways dependent on Mitogen Activated Protein Kinases is sufficient to increase the population of macrophages in the healthy myocardium (26).

## Future directions

Hence, cardiomyocytes and macrophages form an interspersed network and dialogue with each other to profile the multiple processes shaping the cardiac tissue. In pathological settings, the exact roles of resident embryonically derived- and monocyte-derived macrophage subsets on cardiomyocyte homeostasis remain to be fully defined. Of note, the use of strategies designed to inhibit CCR2 signaling or endothelial cell-dependent adhesion is of interest to abrogate monocyte-derived macrophages upregulation but likely induces non-specific effects on other inflammatory cell subtypes and, more importantly, hampers the direct impact of monocytes on cardiac remodeling. Such side effects may lead to confounding outcomes and misinterpretation of cardiac macrophages related-actions. For example, monocyte-derived macrophages likely display both positive and negative potential on cardiomyocyte and cardiac tissue. As such, CD206^+^F4/80^+^CD11b^+^ macrophages exhibit strengthened reparative abilities after myocardial infarction. This macrophage subpopulation emerges from bone marrow HSCs through a kinase TRIB1 dependent pathway (73). In addition, IL-4 administration increases the number of reparative macrophages and enhances the post-myocardial infarction prognosis in mice (73). In the stressed heart, one can also speculate that the inflammatory landscape could skew the status of cardiac-resident macrophages from a reparative to a deleterious phenotype. Therefore, studies using specific resident macrophage depletion or distinct resident macrophage gene deletion need to be developed. Nevertheless, cardiac tissue provides a revealing example where macrophage number and phenotype, duration of macrophage-related signaling, from acute to chronic, could balance the protective and pathogenic transition of cardiac tissue.

Inflammation-dependent remodeling process has been predominantly described in acute injury, such as acute myocardial infarction. However, the impact of these inflammatory signalings remain mostly undefined in chronic pathological settings (74). Notably, further studies need to explore the mechanisms underlying the direct roles of macrophages on cardiomyocyte survival and hypertrophy and if specific macrophage sup-population could regulate the progression from compensated hypertrophy to heart failure. Finally, the precise identification of the molecular and cellular signals send from cardiomyocytes toward macrophages would likely improve our understanding of cardiac macrophage physiology and both their beneficial and deleterious actions.

## Author contributions

IG, VD, and J-SS perform literature search and writing. All authors approved the manuscript.

### Conflict of interest statement

The authors declare that the research was conducted in the absence of any commercial or financial relationships that could be construed as a potential conflict of interest.
